# Commentary: A construct divided: prosocial behavior as helping, sharing, and comforting subtypes

**DOI:** 10.3389/fpsyg.2016.00491

**Published:** 2016-04-07

**Authors:** Bahar Tunçgenç

**Affiliations:** Department of Anthropology, Institute of Cognitive and Evolutionary Anthropology, University of OxfordOxford, UK

**Keywords:** prosocial behavior, socio-cognitive development, emotional development, commentary, early childhood

Research over the decades has shown clearly that human children act pro-socially starting from very early in life. In her recent paper, Dunfield (2014) has contributed to this wealth of research by proposing a framework in which to understand children's pro-social behaviors. Here, I aim to draw attention to some drawbacks of this framework as well as suggest directions for future research.

According to Dunfield's (2014) framework, pro-social behavior comprises of three subtypes: helping, sharing and comforting. The negative state that a pro-social behavior targets is what forms the basis of this categorization. Accordingly, alleviating a negative state marked by an *instrumental need* requires helping behavior, while an *unmet material desire* requires sharing behavior, and *emotional distress* requires comforting (Dunfield, 2014). Identifying these subtypes is helpful for conceptual clarity, for disentangling the socio-cognitive skills underlying pro-social behaviors, and for a complete understanding of the developmental trajectory of pro-sociality.

One concern, however, regards ecological validity. How distinct are the three subtypes of pro-social behavior really? Imagine a daily life event, where a friend loses their wallet. You may respond to their negative state by helping them look for their wallet, by sharing some of your money with them, or by comforting them and showing sympathy. In such real-life examples, the negative states are largely intermeshed and thus, there is often more than one “right” way to respond. This is particularly so when considering the interference of *emotional distress*, which is nearly impossible to detach from *instrumental needs* or *unmet material desires*.

In fact, most empirical studies have incorporated *emotional distress* while testing helping and/or sharing behavior (but see Kenward and Gredebäck, 2013). The widely used out-of-reach instrumental helping tasks (e.g., Warneken and Tomasello, 2006; Over and Carpenter, 2009; Brownell et al., 2013a) rely on linguistic and/or facial cues that indicate distress, such as the experimenter uttering “Oops!” or “Oh!” to mark the accidental nature of the action, and hence, her need for help. Only a few studies (Vaish et al., 2009; Newton et al., 2014; Chiarella and Poulin-Dubois, 2015) have investigated whether the experimenter maintaining a neutral expression in face of a negative event would influence children's subsequent helping behavior. Yet, the emotion expression manipulation in these studies was done for events preceding the pro-social task; the negative emotional cues were still provided during the pro-social task. Hence, although infants acted equally pro-socially in neutral expression conditions, the relative effects of emotional distress and instrumental need on helping behavior remain inseparable.

The high co-occurrence of emotional distress and instrumental need in real-life examples and empirical set-ups reveal how interlinked the two subtypes are. In addition, it suggests that current methods for assessing helping behavior have actually been assessing how children respond to another individual's instrumental need *and* emotional distress combined. Regarding Dunfield's (2014) framework, the existing literature does not permit us to tease apart the emotional and goal-directed influences on children's representation of the problem in helping scenarios. Dunfield (2014) claims that children represent the problem in helping scenarios via inferring the instrumental need from the other person's goals. Yet, the role played by negative emotional cues, e.g., “Oops!,” might be just as crucial in assessing the needy situation of another person.

Studies conducted with children with autism spectrum disorders (ASDs) can be insightful, as children with ASDs can understand the goals of others (Carpenter et al., 2001; Hamilton et al., 2007) but do have significant problems with empathizing (e.g., Baron-Cohen and Swettenham, 1997). Using out-of-reach helping tasks similar to ones used with typically developing children, Liebal et al. (2008) has found that the ASD group displayed significantly less helping behavior than did children with other developmental disorders. It can be reasoned that the ASD group's difficulty in understanding the negative emotional content of the situation, but not difficulty in goal understanding, may have caused the decrease in helping behavior. Yet, future research should test this idea more directly by assessing children's emotion understanding capabilities and controlling the emotional expressions of the helpee.

Although, the examples so far have focused on the link between emotional distress and helping behavior, a similar case can be made for sharing behavior as well. Yet, due to the wider variety of sharing tasks used in different studies, the interference of emotional expressions with sharing behavior is also variable. Still, there seems to be a trend such that studies suggesting an earlier proclivity for sharing involve more overt cues about the emotional distress that the potential recipient is experiencing. For instance, when the potential recipient explicitly asked for the object they wanted and/or indicated an interest in it, as young as 18-month-old children spontaneously shared their resources (e.g., Brownell et al., 2009, 2013b; Dunfield et al., 2011; Schmidt and Sommerville, 2011; Sommerville et al., 2013). On the other hand, when children were asked to allocate resources among unfamiliar, distant or hypothetical others, a preference for equity was reported as emerging only after 6–8 years of age (Fehr et al., 2008; Blake and McAuliffe, 2011; Shaw and Olson, 2012). To distinguish between how much of the sharing behavior observed is due to children's representation of the problem as *unmet material desire* and *emotional distress*, more studies are needed that control for the emotional expressions and/or use inanimate entities as potential recipients (e.g., Sloane et al., 2012).

To conclude, it is recommended that future research take into account the potential effects of emotional distress on children's helping and sharing behaviors. A simple methodological aid may be contrasting conditions where an individual expresses their negative emotions vs. not. Using non-social controls and/or entities that are not expected to display emotions, e.g., puppets, animal figures etc., may also be appropriate. Finally, neuroscientific studies can be informative by revealing which brain areas are recruited (e.g., Paulus et al., 2013). Essentially, controlling for emotional distress would yield more accurate information on the specific socio-cognitive mechanisms required for each subtype of pro-social behavior. It would also increase the explanatory power of Dunfield's (2014) framework in more naturalistic settings.

## Author contributions

The author confirms being the sole contributor of this work and approved it for publication.

### Conflict of interest statement

The author declares that the research was conducted in the absence of any commercial or financial relationships that could be construed as a potential conflict of interest.
